# Conjugative Gene Transfer between Nourished and Starved Cells of *Photobacterium damselae* ssp. *damselae* and *Escherichia coli*

**DOI:** 10.1264/jsme2.ME19099

**Published:** 2019-12-27

**Authors:** Yoshiaki Kohyama, Satoru Suzuki

**Affiliations:** 1 Center for Marine Environmental Studies, Ehime University Matsuyama, Ehime, 790–8577 Japan

**Keywords:** horizontal gene transfer, oligotrophic conditions, starvation, transcription, organic matter

## Abstract

Horizontal gene transfer (HGT) between bacteria with different habitats and nutritional requirements is important for the spread of antibiotic resistance genes (ARG). The objective of the present study was to clarify the effects of organic matter on HGT between nourished and starved bacteria. We demonstrated that conjugation ability is affected by the nutritional conditions of the cell and environment. A filter mating HGT experiment was performed using *Photobacterium damselae* ssp. *damselae*, strain 04Ya311, a marine-origin bacterium possessing the multidrug-resistance plasmid pAQU1, as the donor, and *Escherichia coli* as the recipient. The donor and recipient were both prepared as nutrient-rich cultured and starved cells. Filter mating was performed on agar plates with and without organic nutrients. The transcription of the plasmid-borne genes *tet*(M) and *traI* was quantitated under eutrophic and oligotrophic conditions. The donor *P. damselae* transferred the plasmid to *E. coli* at a transfer rate of 10^−4^ under oligotrophic and eutrophic conditions. However, when the donor was starved, HGT was not detected under oligotrophic conditions. The addition of organic matter to starved cells restored conjugative HGT even after 6 d of starvation. The transcription of *traI* was not detected in starved cells, but was restored upon the addition of organic matter. The HGT rate appears to be affected by the transcription of plasmid-associated genes. The present results suggest that the HGT rate is low in starved donors under oligotrophic conditions, but is restored by the addition of organic matter.

A natural aquatic environment is a reservoir of antibiotic resistance genes (ARGs) (31, 35), in which ARGs may be horizontally transferred among diverse bacterial communities by conjugation, transduction, and transformation. For example, the transferable plasmid pRAS1 may transfer in marine sediments (29), and horizontal gene transfer (HGT) was found to occur between aquatic oligotrophic bacteria and human-related bacteria by class 1 integrons (7). HGT between bacteria having different habitats and nutritional requirements is important in terms of the risk of ARG spreading.

Experimental conjugative HGT between marine bacteria and *Escherichia coli* has been known for more than two decades (*e.g.*, 14, 24). The conjugative HGT rate is controlled by the physiological conditions of the gene donor (9), in which F-pili are lost and pairing between cells does not occur in starved donors. The HGT rate was shown to decrease in a manner that was dependent on the starvation period when mating was performed between *Vibrio* and *E. coli* and also between *Vibrio* strains (14). A recent study examined the effects of organic matter on HGT, with organic matter being identified as a factor that accelerates HGT on a plastic surface (1). Based on previous findings, it is reasonable to hypothesize that organic nutrient conditions in a water environment affect the HGT rate between donor and recipient bacteria.

A natural water environment is basically oligotrophic. Coastal seas are oligotrophic due to a low nutrient supply and dilution, while the concentration of organic carbon may vary widely (6–450 μg L^−1^) (27). The most abundant commensal bacteria in the sea are oligotrophs (18), which constitute a class that markedly differs from enteric and pathogenic bacteria. Although HGT has been examined in aquatic environments, limited information is currently available on the dynamics of HGT events between ARG possessing marine bacteria and human-related bacteria in terms of the nutritional status of cells and the surrounding environment.

We speculate that pathogenic/enteric bacteria may encounter marine bacteria. For example, residual bacteria from sewage treatment plants (STPs) flow into rivers and the sea (35). STPs themselves serve as hot spots for the generation of multidrug-resistant bacteria (17). Thus, bacteria released from STPs may play a role in the spread of ARGs into the environment. ARGs in commensal bacterial communities have the potential to be transferred to *E. coli* and vice versa (25, 28). These environmental ARGs may be capable of invading the human environment from coastal seas in which various bacteria co-exist. Aquaculture products, fishermen, and individuals frequenting recreational areas are candidate ARG carriers (19). Based on the risk of exposure to ARG from aquatic environments, more detailed information is needed on HGT between marine and human bacteria in the sea.

Recent resistome studies revealed that not only ARGs, but also mobile genetic elements may be retained in coastal water and sediments (21, 22, 34), and possibly also in animal farms (20). Thus, a wide range of environments are possible transmission sites as well as reservoirs of ARGs (4, 6).

In the present study, we examined the HGT rate and transcript levels of the plasmid-borne *traI* and *tet*(M) genes in pAQU1 (25), which is a MOB_H_ family plasmid bearing multi-drug resistance that is widely distributed in coastal marine bacteria. Donor and recipient bacteria were cultured in nutrient medium or exposed to nutrient-poor artificial seawater or saline to generate starved cells. A conjugative HGT experiment mimicking exposure to marine and enteric bacteria was designed to quantitate HGT and related gene expression.

## Materials and Methods

### Bacteria and culture

*Photobacterium damselae* ssp. *damselae* strain 04Ya311 was used as the ARG donor (25). This strain possesses the multidrug-resistance plasmid pAQU1, which harbors one copy each of *bla*_CARB-9_-like, *floR*, *mef*(C), *mph*(G), *sul2*, *tet*(M), and *tet*(B). The plasmid also carries 17 *tra* genes, four integrase family genes, and *parA*, *parB*, and *rep* genes, providing transfer capability. *E. coli* strain W3110 was used as the recipient. Strain 04Ya311 and the transconjugant *E. coli* (TJ-W3110) possess pAQU1 as a single copy plasmid (3). *P. damselae* was cultured in LB medium (BD Difco^™^, Sparks, MD, USA) supplemented with 2% NaCl and 20 μg mL^−1^ oxytetracycline (OTC) at 25°C, while *E. coli* was cultured in LB medium at 37°C. Since *P. damselae* is a marine species, medium based on seawater is a better representative of natural physiological conditions. However, in order to provide identical conditions to those used for *E. coli*, LB was only supplemented with NaCl in comparisons with *E. coli*. To examine transcription in *P. damselae*, we used marine broth (MB, BD Difco^™^) to provide growth conditions that were as close as possible to natural salt levels.

### Starvation treatment

Donor and recipient cells were cultured in the LB-based liquid media described above and harvested by centrifugation at 8,400×*g* at 4°C for 10 min. Cells were washed once with artificial seawater (ASW) for *P. damselae* and with phosphate-buffered saline (PBS) for *E. coli* and then suspended in 1 L of ASW (*P. damselae*) or PBS (*E. coli*). The final density of resuspended washed cells was 10^7^ cells mL^−1^. The resulting cell suspension was kept at 25°C (*P. damselae*) or 37°C (*E. coli*) for 6 d. To avoid the generation of organic matter by self-digestion, ASW and PBS were changed 2 and 4 d after resuspension. These starved cells were harvested after 6 d by centrifugation at 15,620×*g* at 4°C for 30 min.

### Cell number and size measurement

Total cells were counted by DAPI staining (3), and live and dead cells were counted by live/dead staining (5) after fixation with 0.3% glutaraldehyde. Colony-forming units (CFUs) were enumerated on plates of LB-1.5% agar plus 2% NaCl (*P. damselae*) or plain LB agar (*E. coli*). Previous studies demonstrated that bacteria grown under starvation conditions were smaller in size (8). We confirmed nutritional effects on cell size under nutritious and starved conditions. Cell sizes were measured with qNano (IZON Science, Christchurch, New Zealand) (13) according to the company’s protocol.

### Conjugation experiments

Four mating pairs of HGT experiments were performed as follows: a nourished donor (D)-nourished recipient (R), nourished donor (D)-starved recipient (r), starved donor (d)-nourished recipient (R), and starved donor (d)-starved recipient (r). Immediately after mixing the donor (1.0×10^6^ cells in 0.5 mL) and recipient (1.0×10^6^ cells in 0.5 mL), the solution was filtered through a nitrocellulose filter (diameter of 25 mm, Merck Millipore, Darmstadt, Germany) with a pore size of 0.22 μm under negative pressure (20 kPa). The filter was washed with 5 mL of PBS to remove medium and then placed on agar medium. To test conjugation under nutrient-rich and -poor conditions, two sets of agar media were prepared: one was a nutrient agar plate (LB), while the second was a PBS+1.5% agar plate. Agar plates were cultured at 25°C for 18 h; the filter was then removed from the agar medium and organisms were suspended in 500 μL of PBS with vortexing. This suspension was diluted 10-fold with PBS and spread on a pair of LB agar plates supplemented with 20 μg mL^−1^ OTC. The first plate was incubated at 42°C. Since *P. damselae* does not grow at this temperature, these conditions were selected for *E. coli* transconjugants (TJ-W3110). The second plate was incubated at 25°C to obtain both the donor and TJ-W3110. The conjugation rate was calculated as TJ-W3110 cells (CFU mL^−1^)/donor+TJ-W3110 cells (CFU mL^−1^). All experiments were performed in triplicate. The presence of pAQU1 in TJ-W3110 was confirmed by performing a PCR reaction targeting *tet*(M). The opposite pairing (donor-*E. coli* TJ-W3110 versus recipient-*P. damselae*) was not performed in the present study; since pAQU1 stably remained in *P. damselae* (3), it was not possible to prepare the pAQU1-free cells of *P. damselae*.

### RNA extraction

An appropriate volume of donor *P. damselae* growing in LB medium and 100 mL of starved cells (prepared as described above) were harvested by centrifugation at 5,000×*g* at 4°C for 10 min. All cultures were grown and harvested in duplicate. Immediately after pelleting cells, RNAprotect bacteria reagent (Qiagen, Hilden, Germany) was added. Total RNA was extracted from the pellets using the RNeasy mini kit (Qiagen) according to the manufacturer’s manual. RNA extraction was performed from triplicate samples. To assess the effects of the addition of organic matter, 100 mL of starved cells was harvested by centrifugation at 5,000×*g* at 4°C for 10 min after 144 h (6 d) of starvation and resuspended in 2 mL of MB containing 5 g peptone and 1 g yeast extract per L. The resulting suspensions were incubated at 25°C for 5 min, 1.5 h, and 4 h. At each time point, 4 mL of RNAprotect bacteria reagent was added to the cultures, and the cell suspension was then centrifuged and total RNA extracted as above.

### mRNA quantification (qPCR)

The total RNA fraction was treated with 5 U of DNase I (Takara, Otsu, Japan) to hydrolyze DNA. Nucleotides were removed by ethanol precipitation. Purified RNA was visually checked on agarose gel and further checked by PCR targeting the *tet*(M) gene, in which amplification products were not formed. RNA amounts were quantified by e-spect (BM Bio, Tokyo, Japan). cDNA was synthesized from 500 ng of the purified RNA fraction using the PrimeScript^™^ 1^st^ strand cDNA Synthesis Kit (Takara). The plasmid-borne genes subjected to transcription quantitation included the *traI* relaxase gene, *tet*(M) resistance gene, and *gyrB* housekeeping gene. *traI* and *tet*(M) were selected because they exist as single-copy genes on pAQU1. Furthermore, *tet*(M) has been studied in detail in HGT and stability in environments (3, 4, 25). *traI* is the initial enzyme acting in the HGT cascade and may be used to monitor *traI* expression, and the HGT rate is appropriate for observing the HGT process. The mRNA levels of these genes in the resulting cDNA were quantitated by real-time PCR with the CFX96 real-time PCR detection system (Bio-Rad, Hercules, CA, USA). Primers for quantitative PCR (qPCR) are listed in Table S1. PCR conditions were as follows: *traI*, 40 cycles at 94°C for 30 s, at 94°C for 15 s, at 59°C for 20 s; *tet*(M), 40 cycles at 95°C for 30 s, at 95°C for 10 s, at 57°C for 20 s; and *gyrB*, 40 cycles at 95°C for 1 min, at 95°C for 10 s, at 62°C for 10 s. Standard curve quality was confirmed within 40 cycles (32, 33).

### Statistical analysis

Statistical variance in conjugation rates and transcript levels was evaluated by the F-test. If variance was homoscedastic, we used the Student’s *t*-test; if not, we used Welch’s *t*-test. In either case, analyses were performed as one-tailed tests. Significant differences were inferred with *P*<0.05 or *P*<0.01.

## Results and Discussion

### Starvation effects on cell physiology

When incubated in ASW for 6 d, the numbers of total, live, and dead *P. damselae* cells and *P. damselae* CFUs decreased by one log, whereas those of *E. coli* fell by two logs (Fig. S1A and B). Fouz *et al.* (11) reported that a one-log decrease in the total cell number of *P. damselae* occurred after 60 d. Their experiment used natural seawater containing low levels of organic matter, which may have extended the survival period. We changed ASW at 2 and 4 d, precluding the accumulation of cell-derived organic matter. The decline in all factors, particularly CFUs, of *P. damselae* was less than that in those of *E. coli*. This result suggested that marine bacteria have a greater ability (16) to adapt to oligotrophic conditions than *E. coli* (23). After 6 d of nutrient deprivation, *P. damselae* exhibited an average cell size of 1128.8±155.0 nm (mean±SD), a significant (*P*<0.01) reduction of 11% from the size of nourished cells (1276.6±143.4 nm) (Fig. S1C). Upon starvation, the size of *E. coli* cells decreased from 1172.9±195.4 nm to 1034.2±115.7 nm, a significant (*P*<0.01) reduction of 11% (Fig. S1D). A previous study reported that the total cell numbers of *P. damselae* and *E. coli* did not change over the course of one month when ASW was not altered during the starvation period (3). In contrast, the present results showed that the strict starvation treatment affected both cell number and size. The minimization of cell size by starvation was confirmed in *P. damselae* and *E. coli*.

### Conjugation rate

**Fig. 1:**
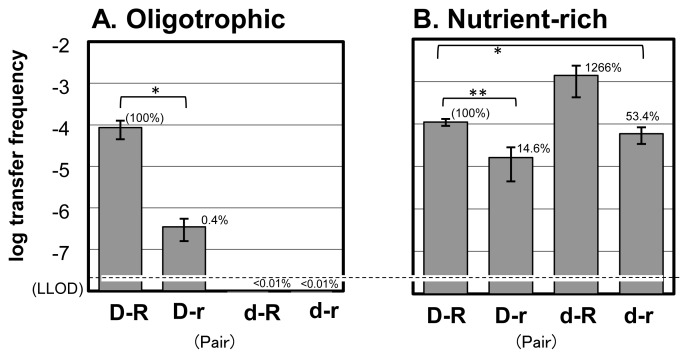
Conjugative gene transfer rates under oligotrophic conditions (A) and nutrient-rich conditions (B). Mating pairs of a gene donor (D, cells cultured in nutrient-rich medium; d, starved cells) and gene recipient (R, cells cultured in nutrient-rich medium; r, starved cells) are shown as D-R, D-r, d-R, and d-r. Values are provided as means±standard deviation (SD) (*n*=3). The dashed line indicates the lower limit of detection (LLOD). The indicated comparisons yielded significant differences of *P*<0.05 (*) and *P*<0.01 (**) by one-tailed Student’s or Welch’s *t*-tests.

The conjugative HGT rate under oligotrophic conditions is shown in Fig. 1A. Transfer frequency in the D-R pair was 8.6×10^−5^, while that in the D-r pair was 3.5×10^−7^ (0.4% of D-R; *P*<0.05). On the other hand, conjugation frequency in pairs with the starved donor, d-R and d-r, was below the limit of detection (<1.0×10^−8^). This result was similar to that previously reported in *Vibrio* (14), which showed that a longer starvation period was associated with a smaller transconjugant number. Fox *et al.* (12) demonstrated that IncP-1 transfer did not increase when nutrients were not replenished. These findings were obtained using IncP plasmids (MOB_P_). Our plasmid, pAQU1, belongs to the MOB_H_ family (25). Since the MOB series have a similar HGT system (30), the conjugation of these MOB plasmids may occur under oligotrophic conditions if the donor is nourished, but not if the donor is starved.

When the conjugation experiment was performed under nutrient-rich conditions (Fig. 1B), high conjugation rates were obtained for all pairs. The conjugation rate with the D-R pair was 1.1×10^−4^, a value that did not differ significantly from that obtained under oligotrophic conditions (*P*=0.20) and was consistent with our previous findings (26). The d-R combination showed a conjugation rate of 1.4×10^−3^; although this value was 12-fold higher than that obtained with the D-R pair, this difference was not significant (*P*=0.07). The rate with the d-r combination was 5.9×10^−5^, a value that was significantly lower (53.4%; *P*<0.05) than that of D-R. This result suggested that supplementation of the medium with organic matter permitted the recovery of the conjugation ability of the starved donor. This result also indicated that if the donor and recipient are both nourished, conjugative HGT may occur even in an oligotrophic environment. When the recipient was starved, the HGT rate decreased (compare D-R and D-r pairs in Fig. 1A and B, respectively). The conditions under which organic matter is supplied to starved cells are expected to resemble those occurring at wastewater effluent points and with aquaculture feeding. These water environments may be HGT hot-spots in a natural aquatic environment. Although HGT from *E. coli* to *P. damselae* was not examined in the present study, we propose HGT from human-derived bacteria to marine bacteria. The dynamics of ARGs between bacteria inhabiting different environments warrant further study.

### Transcription of *traI*, *tet*(M), and *gyrB*

**Fig. 2:**
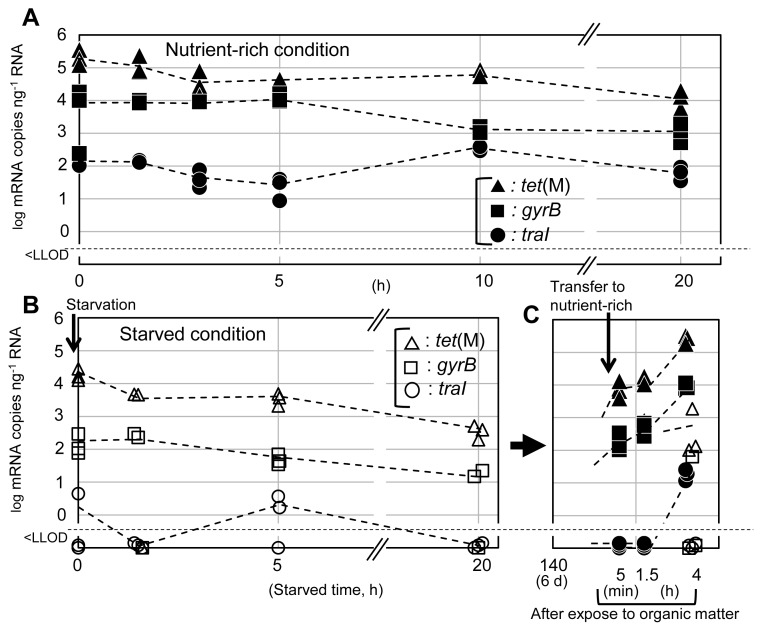
Transcript levels of *tet*(M), *traI*, and *gyrB* genes after 20 h of growth under nutrient-rich (A) and starvation (B) conditions. Panel C shows the effects on transcript levels upon the feeding of starved cells. Continuous starvation was obtained by growth in ASW for 6 d following by shifting to nutrient medium. Transcript levels were obtained as mRNA copy numbers divided by the total RNA amount (ng). Closed symbols show nutrient-rich conditions, and open symbols show starved conditions. Values obtained from triplicate samples are plotted at each time point; means are connected by dotted lines.

Starvation suppresses the expression of F-pili gene (9). In the present study, transcript levels were measured for the *P. damselae* donor plasmid genes *traI*, *tet*(M), and chromosomal *gyrB*. All of these genes were expressed in the nutrient-rich culture (Fig. 2A). *tet*(M) and *gyrB* transcript levels appeared to be higher than those of *traI* throughout the study period. Expression levels were not altered by the addition of OTC (Fig. S2), suggesting that *tet*(M) mRNA accumulates independently of selective pressure. *traI* encodes a relaxase that catalyzes the cleaving-joining reaction at the initiation and termination of the conjugation process (2). Since a given cell possesses only one copy of pAQU1 (3), the lower transcript levels of *traI* than *tet*(M) and *gyrB* may be sufficient to provide TraI functions. When cells were suspended in ASW, transcript levels were lower than those obtained under nutrient-rich conditions (Fig. 2B). Notably, the levels of *traI* transcripts rapidly decreased, falling to undetectable levels after 1 h; in contrast, *tet*(M) and *gyrB* transcripts levels were still detectable after 20 h of starvation. However, *gyrB* did not appear to be expressed after 6 d of starvation. The GyrB protein is known to provide DNA relaxation during the DNA replication process (10). Cell growth stopped during the starvation period (Fig. S1A), suggesting that *gyrB* expression was negatively controlled under starvation conditions. Immediately (within 5 min) after starved cells had been shifted to nutrientrich medium, *tet*(M) and *gyrB* transcripts were detected at similar levels to those at the baseline (at the start of starvation) (Fig. 2C). In contrast, *traI* transcripts only accumulated to detectable levels 4 h after the transfer to nutrient medium. These results indicated that the expression of *tet*(M) and *gyrB* recovers more quickly than that of *traI*. These results may explain why conjugation was not detected with starved donors under oligotrophic conditions, as shown in Fig. 1A: specifically, starvation results in the cessation of the expression of conjugation genes. The addition of organic matter may evoke the expression of conjugation genes, thereby permitting the recovery of conjugation ability. Conjugation occurs through a cascading system, in which relaxase (TraI) is initially nicked at *oriT*, and the plasmid then replicates. The product, single-strand DNA, moves to the recipient cell through type IV secretion proteins (30). The present study showed the expression of *traI* by the addition of organic matter and an increasing HGT rate, suggesting that if the initiation reaction is activated, cascade signaling occurs.

In conclusion, we herein demonstrated that *P. damselae*, an environmental bacterial donor of ARG, provides conjugative HGT to *E. coli* under oligotrophic conditions if donor cells are actively growing. Although the conjugation ability of cells is decreased by starvation, supplementation with organic nutrition restored HGT. Previous studies reported that organic matter increases bacterial colonization on material surfaces (1, 15), on which the cell-to-cell contact frequency is expected to be high. HGT among members of the bacterial community is expected to occur in nutrient inflow environments, and to a lesser extent in oligotrophic environments. The present results provide further insights into HGT in oligotrophic environments.

## SUPPLEMENTARY MATERIAL



## References

[1] Arias-Andres M., Klümper U., Rojas-Jimenez K., Grossart H-P. (2018). Microplastic pollution increases gene exchange in aquatic ecosystems. Environ Pollut.

[2] Balzer D., Pansegrau W., Lanka E. (1994). Essential motifs of relaxase (TraI) and TraG proteins involved in conjugative transfer of plasmid RP4. J Bacteriol.

[3] Bien T.L.T., Sato-Takabe Y., Ogo M., Usui M., Suzuki S. (2015). Persistence of multi-drug resistance plasmids in sterile water under very low concentrations of tetracycline. Microbes Environ.

[4] Bien T.L.T., Thao N.V., Kitamura S-I., Obayashi Y., Suzuki S. (2017). Release and constancy of an antibiotic resistance gene in seawater under grazing stress by ciliates and heterotrophic nanoflagellates. Microbes Environ.

[5] Boulos L., Prévost M., Barbeau B., Coallier J., Desjardins R. (1999). LIVE/DEAD^®^ BacLight^™^: application of a new rapid staining method for direct enumeration of viable and total bacteria in drinking water. J Microbiol Methods.

[6] Cabello F.C., Godfrey H.P., Tomova A., Ivanova L., Dölz H., Millanao A., Buschmann A.H. (2013). Antimicrobial use in aquaculture re-examined: its relevance to antimicrobial resistance and to animal and human health. Environ Microbiol.

[7] Chakraborty R., Kumar A., Bhowal S.S., Mandal A.K., Tiwary B.K., Mukherjee S. (2013). Diverse gene cassettes in class 1 integrons of facultative oligotrophic bacteria of River Mahananda, West Bengal, India. PLoS One.

[8] Chien A-C., Hill N.S., Levin P.A. (2012). Cell size control in bacteria. Curr Biol.

[9] Curtiss R., Caro L.G., Allison D.P., Stallions D.R. (1969). Early stages of conjugation in *Escherichia coli*. J Bacteriol.

[10] Filutowicz M. (1980). Requirement of DNA gyrase for the initiation of chromosome replication in *Escherichia coli* K-12. Mol Gen Genet.

[11] Fouz B., Toranzo A.E., Marco-Noales E., Amaro C. (1998). Survival of fish-virulent strains of *Photobacterium damselae* subsp. *damselae* in seawater under starvation conditions. FEMS Microbiol Lett.

[12] Fox R.E., Zhong X., Krone S.M., Top E.M. (2008). Spatial structure and nutrients promote invasion of IncP-1 plasmids in bacterial populations. ISME J.

[13] Garza-Licudine E., Deo D., Yu S., Uz-Zaman A., Dunbar W.B. (2010). Portable nanoparticle quantization using a resizable nanopore instrument-The IZON qNano^™^.

[14] Goodman A.E., Hild E., Marshall K.C., Hermansson M. (1993). Conjugative plasmid transfer between bacteria under simulated marine oligotrophic conditions. Appl Environ Microbiol.

[15] Grossart H-P., Kiørboe T., Tang K., Ploug H. (2003). Bacterial colonization of particles: growth and interactions. Appl Environ Microbiol.

[16] Kaberdin V.R., Montánchez I., Parada C., Orruño M., Arana I., Barcina I. (2015). Unveiling the metabolic pathways associated with the adaptive reduction of cell size during *Vibrio harveyi* persistence in seawater microcosms. Microb Ecol.

[17] Karkman A., Do T.T., Walsh F., Virta M.P.J. (2018). Antibiotic-resistance genes in waste water. Trends Microbiol.

[18] Lauro F.M., McDougal D., Thomas T. (2009). The genomic basis of trophic strategy in marine bacteria. Proc Natl Acad Sci USA.

[19] Leonard A.F.C., Zhang L., Balfour A.J., Garside R., Gaze W.H. (2015). Human recreational exposure to antibiotic resistant bacteria in coastal bathing waters. Environ Int.

[20] Mazurek J., Bok E., Baldy-Chudzik K. (2018). Complexity of antibiotic resistance in commensal *Escherichia coli* derived from pigs from an intensive-production farm. Microbes Environ.

[21] Muziasari W.I., Managaki S., Pärnänen K., Karkman A., Lyra C., Tamminen M., Suzuki S., Virta M. (2014). Sulphonamide and trimethoprim resistance genes persist in sediments at Baltic Sea aquaculture farms but are not detected in the surrounding environment. PLoS One.

[22] Muziasari W.I., Pitkänen L.K., Sørum H., Stedfeld R.D., Tiedje J.M., Virta M. (2017). The resistome of farmed fish feces contributes to the enrichment of antibiotic resistance genes in sediments below Baltic Sea fish farms. Front Microbiol.

[23] Na S.H., Miyanaga K., Unno H., Tanji Y. (2006). The survival response of *Escherichia coli* K12 in a natural environment. Appl Microbiol Biotechnol.

[24] Neela F.A., Nonaka L., Rahman M.H., Suzuki S. (2009). Transfer of the chromosomally encoded tetracycline resistance gene *tet*(M) from marine bacteria to *Escherichia coli* and *Enterococcus faecalis*. World J Microbiol Biotechnol.

[25] Nonaka L., Maruyama F., Miyamoto M., Miyakoshi M., Kurokawa K., Masuda M. (2012). Novel conjugative transferable multiple drug resistance plasmid pAQU1 from *Photobacterium damselae* subsp. *damselae* isolated from marine aquaculture environment. Microbes Environ.

[26] Nonaka L., Maruyama F., Onishi Y., Kobayashi T., Ogura Y., Hayashi T., Suzuki S., Masuda M. (2014). Various pAQU plasmids possibly contribute to disseminate tetracycline resistance gene *tet* (M) among marine bacterial community. Front Microbiol.

[27] Poindexter J.S., Schaechter M. (2009). Low-nutrient environments. Encyclopedia of Microbiology.

[28] Razavi M., Marathe N.P., Gillings M.R., Flach C-F., Kristiansson E., Larsson D.G.J. (2017). Discovery of the fourth mobile sulfonamide resistance gene. Microbiome.

[29] Sandaa R-A., Enger O. (1994). Transfer in marine sediments of the naturally occurring plasmid pRAS1 encoding multiple antibiotic resistance. Appl Environ Microbiol.

[30] Smillie C., Garcillán-Barcia M.P., Francia M.V., Rocha E.P., de la Cruz F. (2010). Mobility of plasmids. Microbiol Mol Biol Rev.

[31] Suzuki S., Hoa P.T.P. (2012). Distribution of quinolones, sulfonamides, tetracyclines in aquatic environment and antibiotic resistance in Indochina. Front Microbiol.

[32] Suzuki S., Ogo M., Miller T.W., Shimizu A., Takada H., Siringan M.A.T. (2013). Who possesses drug resistance genes in the aquatic environment?: sulfamethoxazole (SMX) resistance genes among the bacterial community in water environment of Metro-Manila, Philippines. Front Microbiol.

[33] Suzuki S., Makihara N., Kadoya A. (2018). Tetracycline resistance gene *tet*(M) of a marine bacterial strain is not accumulated in bivalves from seawater in clam tank experiment and mussel monitoring. Sci Total Environ.

[34] Tamminen M., Karkman A., Lõhmus A., Muziasari W.I., Takasu H., Wada S., Suzuki S., Virta M. (2011). Tetracycline resistance genes persist at aquaculture farms in the absence of selection pressure. Environ Sci Technol.

[35] Zhang X-X., Zhang T., Fang H.H.P. (2009). Antibiotic resistance genes in water environment. Appl Microbiol Biotechnol.

