# Supplementary material for: Conjugative Gene Transfer between Nourished and Starved Cells of Photobacterium damselae ssp. damselae and Escherichia coli
Source: Microbes Environ. 2019 Dec 27;34(4):388–92. doi: 10.1264/jsme2.ME19099 (PMC6934395; doi:10.1264/jsme2.ME19099)
Supplement: Supplementary file 1 [file 34_388_s1.pdf]

Table S1. Primers used in this study

Target gene	Sequence (5'-> 3')	Reference
tet(M)	Forward: GCAATTCTACTGATTTCTGC<br>Reverse: CTGTTTGATTACAATTTCCGC	Tamminen et al. (2011) (34)
traI	Forward: AGAGGTAGTAGCTTCCCAGGTTA<br>Reverse: GGCATGACTAAACGGTCGTACTC	This study
gyrB	Forward: GTGGCAATGCAGTGGAATGA<br>Reverse: CGCATCATCACCAGAAGTCG	This study

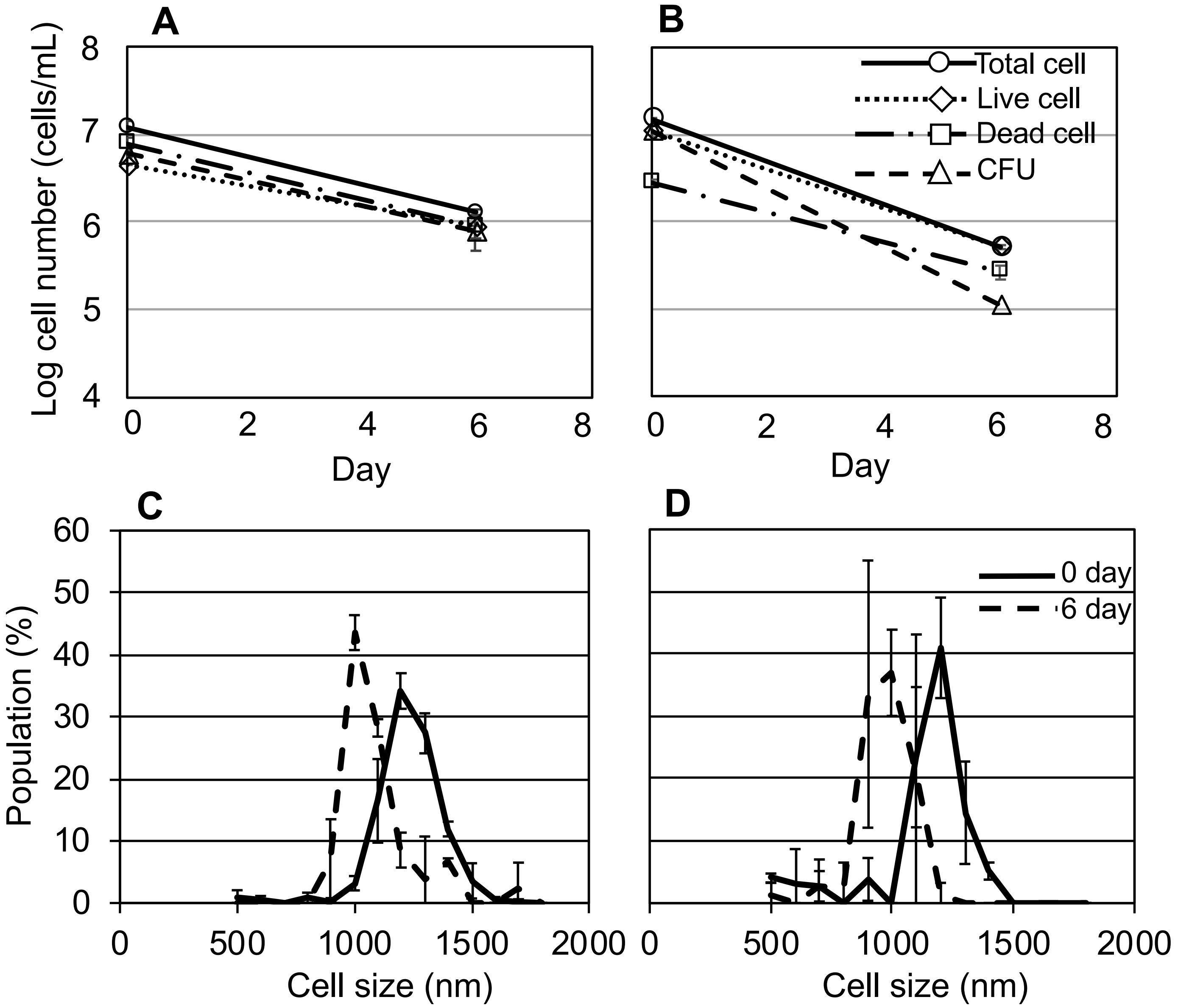

Fig. S1. Starvation effect on cell number (A, B) and cell size (C, D). A, cell numbers of Photobacterium damselae 04Ya311; B, cell numbers of E. coli W3110. Total cell number was determined by counting DAPI-stained cells, live and dead cell numbers were determined by counting cells following live/dead staining, and colony-forming units (CFUs) were determined by enumerating colonies following spreading on agar plates. Cell numbers at 0 days and 6 days are provided as mean ± standard deviation (SD) from triplicate experiments; note that SD bars in this plot are typically obscured by symbols themselves. C, cell size distribution of 04Ya311; D, cell size distribution of W3110. Population percentages (provided as mean ± SD) were obtained from measurement of 355 cells (04Ya311, 0 days), 43 cells (04Ya311, 6 days), 76 cells (W3110, 0 days) and 50 cells (W3110, 6 days).

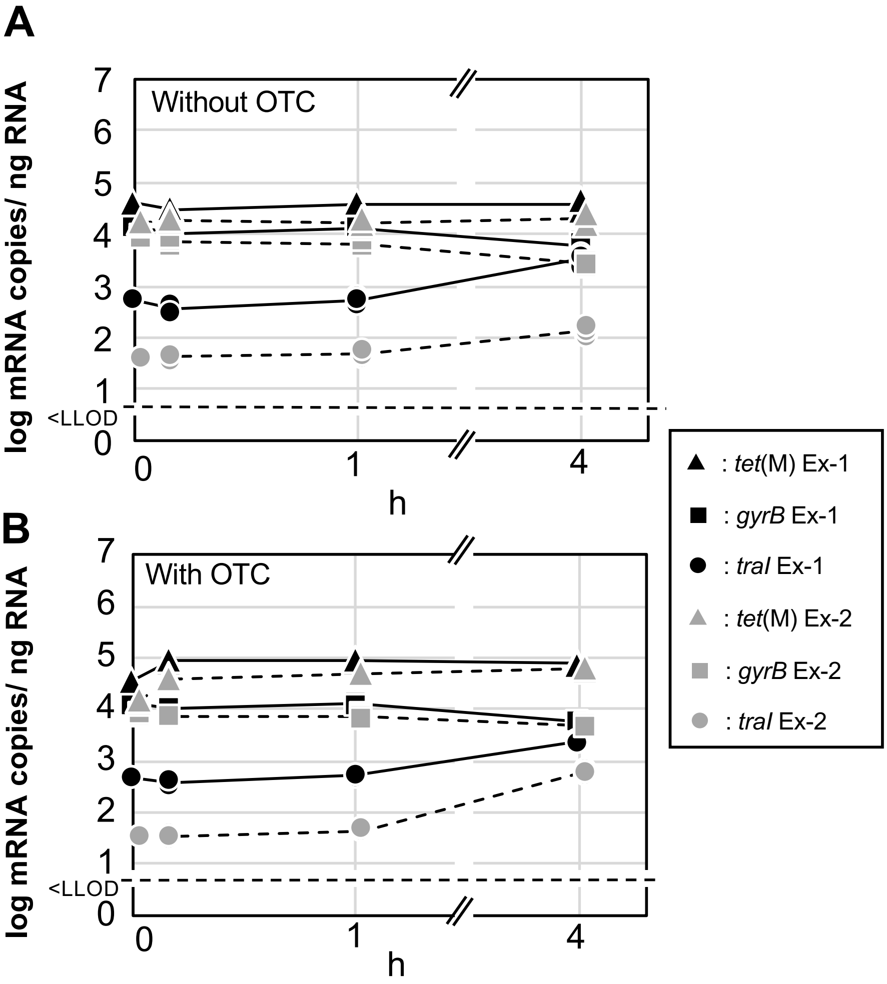

Fig. S2. Transcript levels under nutrient-rich conditions without (A) or with 60 μg/mL of oxytetracycline (B). This experiment was performed two times (black symbols, 1st experiment; grey symbols, 2nd experiment). Each symbol is the mean value from duplicate samples. Dashed line indicates the lower limit of detection (LLOD).
